# Surgical removal of a peripheral venous catheter fragment in the heart in a preterm infant

**DOI:** 10.1186/s13019-024-02818-4

**Published:** 2024-05-31

**Authors:** Gang Wang, Zhe Zhao, Gengxu Zhou, Zhichun Feng

**Affiliations:** 1 The Second School of Clinical Medicine, Southern Medical University, Guangzhou, 510515 China; 2 Department of Pediatric Cardiac Surgery, The Seventh Medical Center of the PLA General Hospital, Beijing, China; 3 Department of Pediatrics, The Seventh Medical Center of the PLA General Hospital, No.5 Nanmencang Road, Dongcheng District, Beijing, 100700 China

**Keywords:** Peripheral venous catheter, Catheter fragment, Foreign body, Case report

## Abstract

Peripheral venous catheter fracture with cardiovascular embolization is a rare but potentially serious complication. Herein, we report a case of peripheral venous catheter fracture with embolization in right ventricle in a preterm infant. The catheter fragment was successfully removed by surgical procedure via median sternotomy under cardiopulmonary bypass(CPB).We hope this case will increase awareness of this rare complication and improve cannulation safety.

**Supplementary Information:**

The online version contains supplementary material available at 10.1186/s13019-024-02818-4.

## Introduction

Peripheral venous cannulation is a widespread and routed medical procedure. Although it is generally a safe procedure, it may cause some common complications such as phlebitis, infiltration, occlusion and dislodgement [1]. Peripheral venous catheter fracture with cardiovascular embolization is a very rare but potentially serious complication. The embolized catheter fragment could cause arrhythmia, pulmonary symptoms, septic syndrome, valve insufficiency, thrombosis, and even cardiac perforation [2, 3]. Herein, we report a case of peripheral venous catheter fracture with embolization in right ventricle in a preterm infant. The catheter fragment was successfully removed by surgical procedure under CPB.

## Case report

**Fig. 1:**
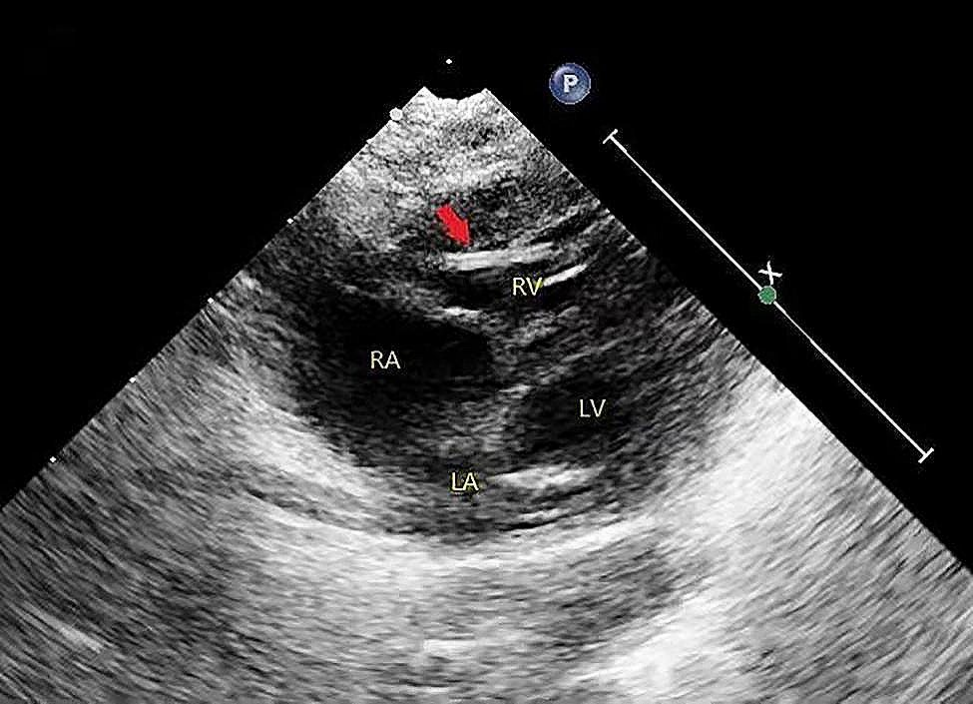
Echocardiography demonstrated a foreign body of 15 mm with line-like strong echo, which was close to the anterior wall of the right ventricle

**Fig. 2:**
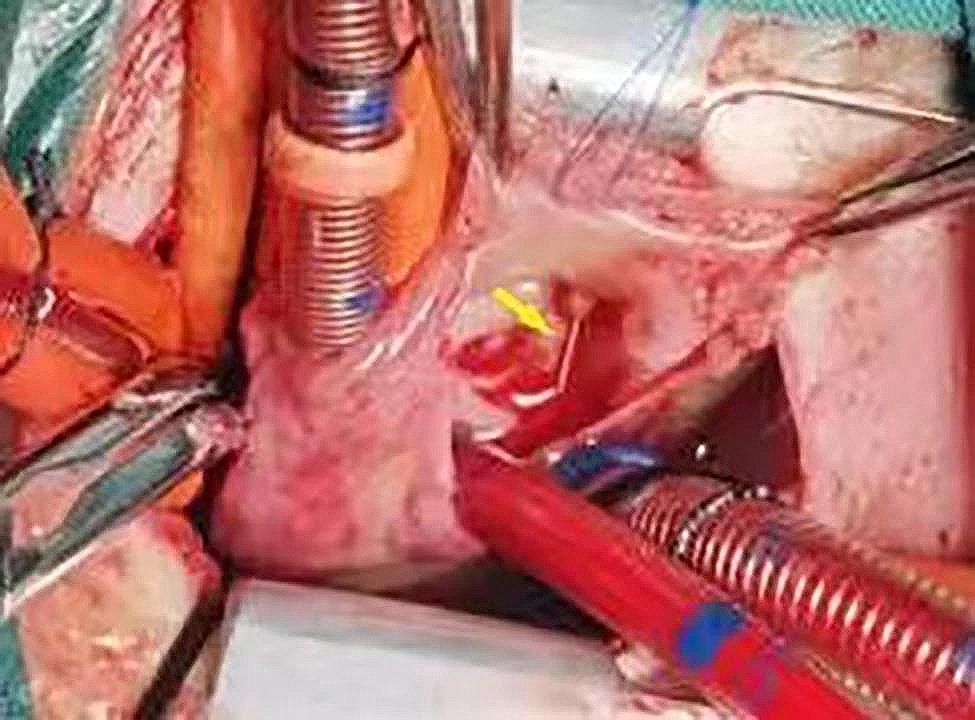
The catheter fragment (yellow arrow) was found trapped in the chordaes of anterior leaflet, and one tip of the fragment was in the right ventricle and the other in the right atrium

**Fig. 3:**
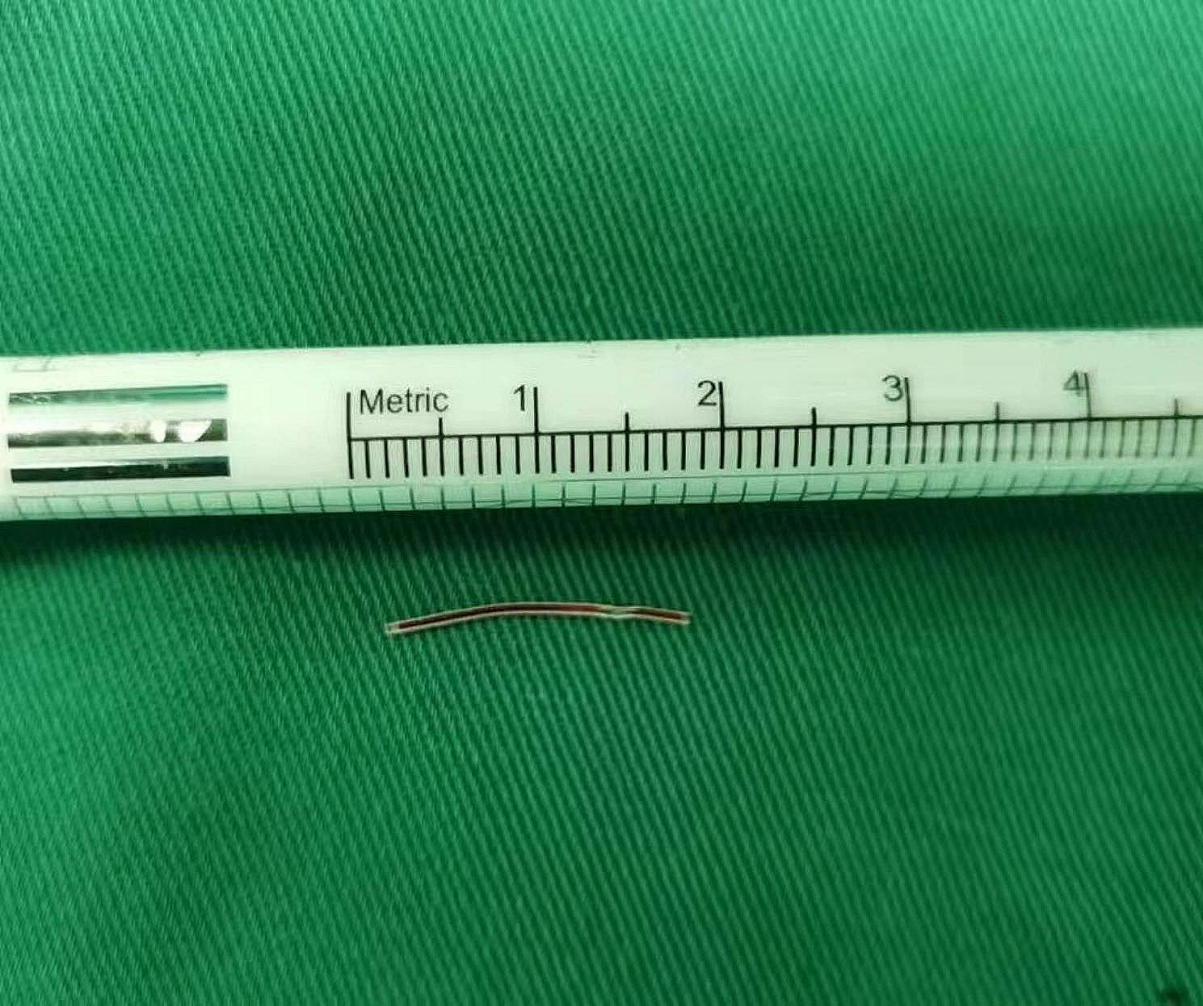
The embolized catheter fragment was removed

A 1-day-old female born after a 34-week gestation with a birth weight of 2.8 kg was admitted to the local hospital due to pneumonia. A peripheral venous catheter was planned to place in the right median cubital vein for medication and intravenous fluids. During cannulation, the catheter accidentally fractured and the distal fragment migrated into the vein. An immediate vascular ultrasound showed no fragment in the upper extremity veins. The patient was immediately transferred to our hospital.The echocardiography demonstrated a foreign body of 15 mm line-like strong echo in the right ventricle, which was close to the anterior wall of the right ventricle (Fig. 1, video 1), with a 2.7 mm patent ductus arteriosus(PDA) and a patent foramen ovale (PFO). However, chest X-ray could not clearly show the catheter fragment. Although the patient was asymptomatic, the embolized catheter fragment in the cardiac chamber may lead to serious complications and require prompt removal. Because the plastic catheter fragment did not have radio opaque substance, it could not be shown by X-ray, percutaneous retrieval under fluoroscopy guidance was not feasible.We had also considered ultrasound-guided percutaneous retrieval, but the catheter fragment was small and attached to the anterior wall of the right ventricle, so it was difficult to snare. The surgical removal of the catheter fragment was performed via median sternotomy with CPB established by aortobicaval cannulation, and the ductus arteriosus was ligated. After the aorta was cross-clamped, the right atrium was opened. The catheter fragment was found trapped in the chordaes of the anterior leaflet, and one tip of the fragment was in the right ventricle and the other side in the right atrium (Fig. 2). We removed the catheter fragment (Fig. 3) and closed the foramen ovale.The postoperative recovery was uneventful.The patient was discharged 12 days after operation and no complication. During the 18 months follow-up, the patient was asymptomatic.

## Discussion

Cardiovascular embolization of iatrogenic catheter fragment is an uncommon but well-known complication. The incidence of this complication varies from 0.2 to 4.2% [3]. This kind of embolization mainly occur with port catheter fragments and peripherally inserted central catheters [3, 4], peripheral venous catheters are very rare, only a few case reports of this complication [5, 6].The guide needle reinsertion was described as the most common mechanism for peripheral venous catheter fracture [5, 7]. In this way, guide needle into an already advanced plastic sheath. The sheath might be curved due to the insertion angle or the trajectory of veins. Advancing the needle may partially or entirely transect the plastic sheath. We speculated that our case was probably above mechanism and was related to an improper procedure. The most common site of embolization is the pulmonary artery followed by the right atrium, right ventricle and the superior vena cava or peripheral vein [3, 8]. In our case, the catheter fragment was trapped in the chordaes of the anterior leaflet, and one tip of the fragment was in the right ventricle and the other in the right atrium.

Clinical symptoms of catheter embolization included catheter malfunction, arrhythmia, pulmonary symptoms, septic syndrome, valve insufficiency, thrombosis, and even cardiac perforation. Meanwhile, there were 24.2% of cases were asymptomatic [3]. Children presented higher rates of asymptomatic cases and septic symptoms [4]. In most cases, the foreign body removed as early as possible, but in a few cases, conservative management could be performed according to the symptoms, size and location of the foreign body, life expectancy and other factors [9, 10].In this case, the patient was a preterm infant, and the catheter fragment was in the right heart chamber with PDA and PFO. Even if the patient was asymptomatic, the risks of mural thrombosis and infective endocarditis remained a threat, and there was a potential risk of catheter fragment migrating into the systemic circulation through PFO or PDA. Rothman reported a case of umbilical venous catheter fragment crossed through PFO with the distal end in the left atrium [11]. Based on the above risks, we planned to remove the catheter fragment promptly.

The methods of retrieving catheter embolization include surgical procedure [5] and percutaneous retrieval [12–14]. A systematic review found that 93.5% of embolized catheter fragments were retrieved percutaneously, only 2.3% of cases need surgical removal [3]. Pazinato and colleagues reported that the success rate of percutaneous retrieval of embolized fragments in children was 96.6% [4]. In this case,. Based on the size and location of the catheter fragment, and it was not be shown by X-ray, we evaluated the percutaneous approach was not feasible.Finally, we chose surgical procedure.

## Conclusion

Peripheral venous catheterization is a widespread medical procedure. Despite this procedure is generally simple and safe, it is essential to prevent the complication of catheter fractureand catheter fragment embolization.Repeated needle reinsertion should be avoided during cannualtion, and the integrity of the catheter should be carefully checked when it was removed. .

### Electronic supplementary material

Below is the link to the electronic supplementary material.

Supplementary Material 1: Transcription of video: Video 1. Echocardiography demonstrated a foreign body of 15 mm with line-like strong echo, which was close to the anterior wall of the right ventricle

## Data Availability

No datasets were generated or analysed during the current study.
